# Adult asthma associated with roadway density and housing in rural Appalachia: the Mountain Air Project (MAP)

**DOI:** 10.1186/s12940-023-00984-x

**Published:** 2023-03-27

**Authors:** W. Jay Christian, John Flunker, Beverly May, Susan Westneat, Wayne T. Sanderson, Nancy Schoenberg, Steven R. Browning

**Affiliations:** 1 Department of Epidemiology and Environmental Health, College of Public Health, The University of Kentucky, 111 Washington Ave, Lexington, KY 40536 USA; 2 Department of Biosystems and Agricultural Engineering, College of Agriculture, Food, and Environment, University of Kentucky, Lexington, KY USA; 3 Behavioral Science, College of Medicine, University of Kentucky, Lexington, KY USA

**Keywords:** Asthma, Adult, Appalachia, Mining, Epidemiology

## Abstract

**Background:**

Appalachian Kentucky is a rural area with a high prevalence of asthma among adults. The relative contribution of environmental exposures in the etiology of adult asthma in these populations has been understudied.

**Objective:**

This manuscript describes the aims, study design, methods, and characteristics of participants for the Mountain Air Project (MAP), and focuses on associations between small area environmental exposures, including roadways and mining operations, and lifetime and current asthma in adults.

**Methods:**

A cohort of residents, aged 21 and older, in two Kentucky counties, was enrolled in a community-based, cross-sectional study. Stratified cluster sampling was used to select small geographic areas denoted as 14-digit USGS hydrologic units (HUCs). Households were enumerated within selected HUCs. Community health workers collected in-person interviews. The proximity of nearby active and inactive coal mining operations, density of oil and gas operations, and density of roadways were characterized for all HUCs. Poisson regression analyses were used to estimate adjusted prevalence ratios.

**Results:**

From 1,459 eligible households contacted, 1,190 individuals were recruited, and 972 persons completed the interviews. The prevalence of lifetime asthma was 22.8%; current asthma was 16.3%. Adjusting for covariates, roadway density was positively associated with current asthma in the second (aPR = 1.61; 95% CI 1.04–2.48) and third tertiles (aPR = 2.00; 95% CI 1.32–3.03). Increased risk of current asthma was associated with residence in public, multi-unit housing (aPR = 2.01; 95% CI 1.27–3.18) compared to a residence in a single-family home. There were no notable associations between proximity to coal mining and oil and gas operations and asthma prevalence.

**Conclusions:**

This study suggests that residents in rural areas with higher roadway density and those residing in public housing units may be at increased risk for current asthma after accounting for other known risk factors. Confirming the role of traffic-related particulates in producing high asthma risk among adults in this study contributes to the understanding of the multiple environmental exposures that influence respiratory health in the Appalachia region.

## Introduction

There are many potential environmental exposures in rural mining communities that raise concerns for respiratory health outcomes. Contributions from both indoor and outdoor environments are potentially significant contributors to respiratory disease. Surface coal-mining operations, including mountaintop removal (MTR) mines, emit atmospheric particulate matter (PM) to surrounding areas. The air quality impacts from these forms of mining have been a community concern since soil, mineral dust, emissions from diesel equipment and blasting, and wind-driven re-suspension of PM may all contribute to ambient PM [1, 2]. Traffic-related pollutants, which differ in composition from mining-related pollutants, may play a role in respiratory disease, but these pollutants have been understudied in rural areas [3]. Further, PM exposures may be influenced by the characteristics of the homes, personal activity patterns, and local topography [4]. Finally, cigarette smoking and the resulting environmental tobacco smoking exposures (ETS) in the homes of rural Appalachian residents have been well-documented [5]. Understanding the impacts of these multiple exposures on asthma and other respiratory outcomes is critical to designing appropriate interventions.

Epidemiologic studies have demonstrated associations between PM and respiratory symptoms and new-onset asthma, asthma hospitalizations, emergency department visits, and deaths [6–9]. Penttinen et al. (2006) reported fine particles are associated with respiratory morbidity in adults with asthma, with the strongest associations between ultrafine and fine particles and decreased lung function [10]. The sources of ambient PM in mining communities could potentially include coal haul roads, blasting operations, meteorological conditions (carried by the wind) or some combination of multiple exposure sources. Additionally, explosives used in the mining process to remove coal overburden contain ammonium nitrate and diesel fuel and release CO_2_, CO, NO, SO_2_, and ammonia during combustion [2, 11]. Studies examining traffic-related air pollutants in adults have shown positive associations with both asthma prevalence and current asthma [12–14].

Residents of Central Appalachia experience the nation’s highest rates of serious respiratory disease [15, 16]. In addition to high rates of adult smoking [17], regional epidemiologic studies suggest associations between adverse respiratory health outcomes and residence in coal mining areas [18–20]. In an ecological analysis, Hendryx and Ahern found that counties in West Virginia with annual coal production of 4 million tons or greater had higher adjusted odds of chronic obstructive pulmonary disease (COPD), lung disease, and other health outcomes in comparison to persons residing in counties with lower coal production levels [19]. Cross-sectional data from 892 adults indicated current asthma prevalence in a coal mining community in Appalachian Kentucky was 18.2%; COPD was estimated at 25.9% [18]. The adjusted prevalence ratio for those who self-reported current asthma was 1.68 (95% CI: 1.11–2.54) and for COPD was 2.47 (95% CI: 1.62–3.74) in communities with mountaintop removal coal mining in comparison to referent communities without mining [18]. These rates exceed national prevalence estimates for the general population by over two and four-fold, respectively.

The body of research by Hendryx et al. has attracted substantial interest from researchers as well as residents of mining communities. In addition to reported associations between communities with potential mining-related exposures, especially surface and mountaintop mining operations, he and allied researchers have found associations between residence in “primary mining communities” and cardiovascular disease [21, 22], birth defects [23], cancers [24, 25], and general health status [19, 20]. These results call into question the specificity of these associations resulting from discrete environmental pollutants, which may be associated with mining. The separation of the confounding and mediating effects of socioeconomic status has been debated and proposed as alternative explanations for these findings [26]. Most of these studies have been ecologic in design or cross-sectional investigations premised on convenience samples, with ecologic measures of exposure. Studies have been limited by an absence of individual-level, quantitative exposure data of dust levels and other environmental exposures (e.g., motor vehicles, ambient pollution) or small area exposure estimates. In addition, these studies have generally lacked adequate control for critical behavioral risk factors (e.g., smoking, diet, physical activity) and social determinants (e.g., lower socioeconomic status, occupation, age, gender) of respiratory diseases like asthma.

While potential environmental exposures from mining operations, roadways, and other emissions sources have attracted concern, lifestyle factors including the high prevalence of cigarette smoking, exposure to environmental tobacco smoke (ETS), housing conditions including dampness and mold exposures, lack of physical activity, and obesity rates are also likely to impact adult asthma prevalence. For example, roughly one-third of adults in this region are current smokers, and an estimated 65% of adults have ever lived with someone who smokes [17, 27]. Few previous studies have closely considered the factors related to housing condition and housing type (single-family, mobile home, apartment), which may influence adult asthma symptoms and episodes [28].

The Mountain Air Project (MAP) is a community-based participatory research project which was designed to investigate the extent, nature, and source of respiratory health inequities in two counties in southeastern Kentucky through a community-engaged assessment of environmental and individual-level exposures. In addition, the study was undertaken to develop an environmental health action strategy to address community-identified exposures of concern for respiratory diseases, including asthma and COPD, and implement and evaluate a community-based intervention [29]. A study goal was to enhance the collection of environmental exposure data for small geographic units and obtain individual-level data on risk factors for several respiratory disease outcomes. The specific aims of this paper are to describe the sampling and design characteristics of the Mountain Air Project (MAP) and the baseline characteristics of the cohort. Further, we present the results of the associations of several small area metrics of exposures to traffic, mining, and oil and gas operations with lifetime and current asthma while adjusting for other asthma risk factors. While the MAP study focuses on several respiratory conditions, this paper presents results only for lifetime and current asthma.

## Methods

A cross-sectional epidemiologic survey was designed and implemented as a component of the Mountain Air Project (MAP) to meet the multiple objectives described above. For the survey, sampling and interviewing of residents would occur on-site in the community with face-to-face interviews. The survey was developed to include a broad range of community-identified exposures of concern, including those from mining, traffic and roadways, and oil and gas operations. In an effort to address some of the limitations of previous ecologic studies [26], the survey also included home environmental exposures and lifestyle risk factors by obtaining individual-level exposure data related to asthma prevalence.

### Study area and population

Our study focused on two Appalachian Kentucky counties (Letcher and Harlan) with long histories of intensive resource extraction and economic disadvantage. This area has the nation’s highest burden of respiratory disease [30], as well as environmental justice concerns stemming from airborne contaminant exposures. The study area was selected based on the presence of extractive industries, marked disparities in respiratory disease, community concerns regarding exposure from coal mining and other extractive industries, and existing infrastructure for mobilizing the project from previous community-based health research [31–33]. In addition, community stakeholders suggested using "hollows" (or hydrological units) as the most relevant geographic unit for defining “neighborhoods” for the cross-sectional study and for ambient air sampling. This approach was discussed in a previous publication [29]. The study was approved by the University of Kentucky Medical Institutional Review Board, and written informed consent was obtained from all participants.

### Eligibility

Inclusion criteria included non-institutionalized, English-speaking adults age 21 or older, of any race or ethnicity, residing within an eligible household in either Letcher or Harlan counties. Eligible households consisted of single-family residences, apartment housing, or mobile homes. One adult participant was recruited per household. If an adult in the household reported having asthma, COPD, coal worker’s pneumoconiosis (black lung), or other respiratory health condition, he or she was encouraged to serve as the participant for that household. If the person with respiratory disease declined to participate and another adult household member without a respiratory condition was eligible, then that person was recruited for the study.

### Geographic site selection and characterization

**Fig. 1:**
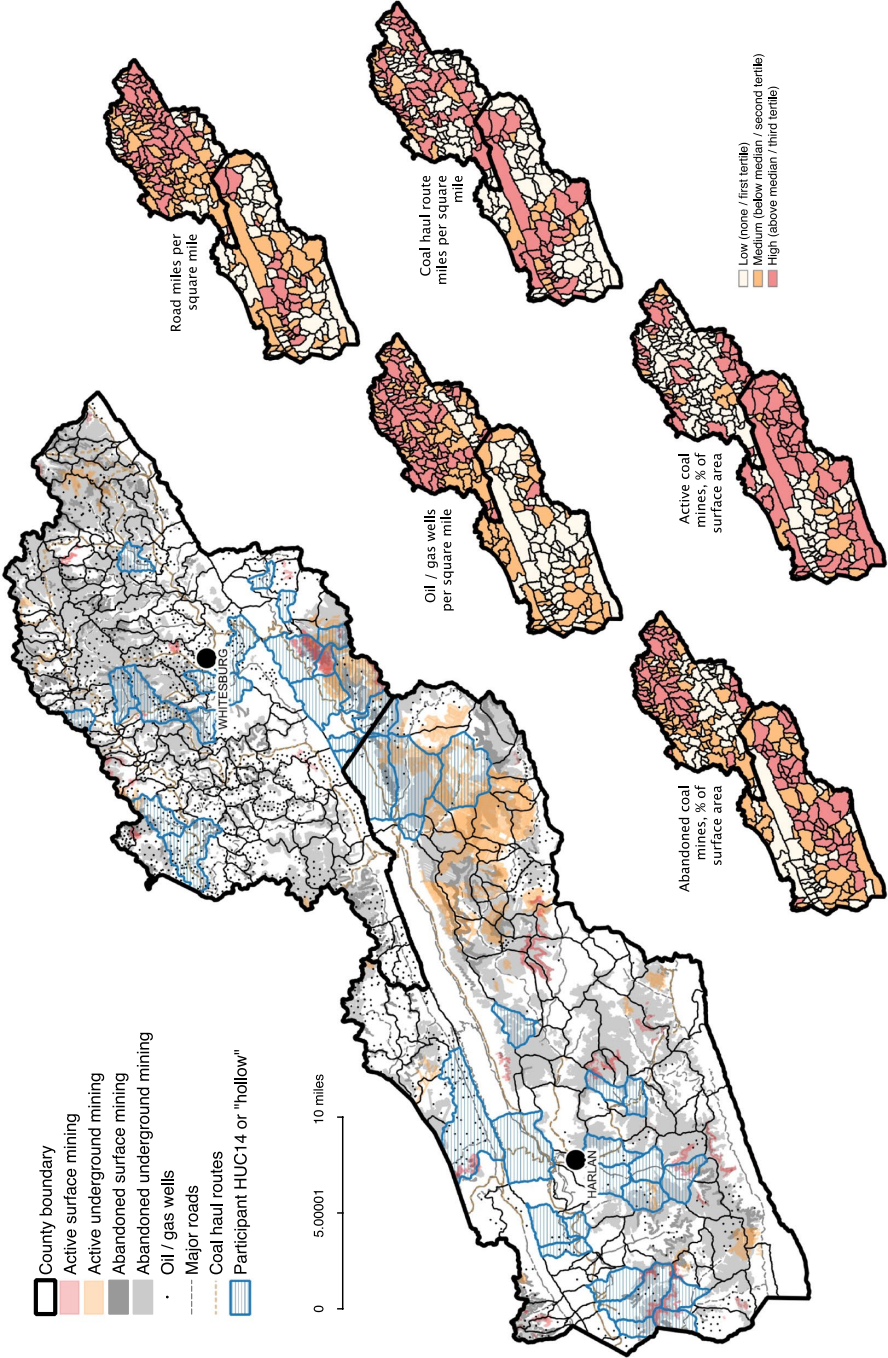
Geographic Distribution of Study HUCs (“Hollows”) and Small Area Exposure Metrics (14 digit HUC) for Harlan and Letcher Counties, Kentucky

We used a stratified cluster sampling technique to select small geographic areas in Harlan and Letcher counties to be the sampling units. We defined candidate ‘hollows’ using GIS map layers representing the boundaries of 14-digit hydrologic unit codes (HUCs). These are the smallest hydrologic units available and often coincide with residential development patterns in the study region since streets and homes are typically ordered in a linear fashion along narrow valleys. We obtained the GIS data for these HUCs from the Kentucky Geological Survey. We imported the HUC boundary polygons into ArcGIS 10.3 [34] and characterized the HUCs by their relationship to several other layers that indicate potential sources of airborne particulates and pollution. These sources of pollutants included the surface boundaries for (a) active underground and surface coal mining sites, and (b) inactive underground and surface coal mining sites, from the Kentucky Mine Mapping Information System; (c) all streets and roads, and (d) roads officially designated as ‘coal haul routes’, from the Kentucky Transportation Cabinet, and (e) the point locations of active oil and gas wells within the HUC, from the Kentucky Geologic Survey. All data sets were the most recent available as of July 2015, just before participant recruitment. Figure 1 displays the distribution of these small area metrics in the study area. From these map layers, we calculated the following metrics to characterize each HUC in ArcGIS:1) abandoned mining (surface or underground), as a percent of HUC total surface area, 2) active mining (surface or underground), as a percent of HUC total surface area, 3) road miles per square mile, 4) coal haul route miles per square mile, and 5) oil and gas wells per square mile. We then summed these ordinal values to create an index of overall environmental risk to respiratory health. The index was divided into tertiles of high, medium, and low presumptive exposure levels to the five sources of airborne particulates above. Ten hollows per each index level were then randomly selected for a total of 30 HUCs included in the study.

Homes in each HUC were enumerated on the ground by field staff. Within each selected HUC, homes were sampled by dividing the total number of homes by an appropriate factor to yield at least ten homes per HUC for the study. Eligible homes were selected using a random starting point and a systematic sample of every n^th^ home. Each selected home had its GPS coordinates recorded using a QStarz GPS data logger. The GPS data were linked with the household survey data and the environmental sampling data so that we were able to develop maps and integrate other datasets with the final epidemiologic files. HUCs with insufficient residences to yield at least ten eligible households were eliminated from the sample, and randomly selected replacement HUCs were provided to the field staff. Ten replacement HUCs were identified through a two-step process that included random selection followed by a rooftop survey using Google satellite imagery. Therefore, although 40 HUCs (hollows) were selected in the sampling process, 30 HUCs with sufficient populations were used in data collection, which was halted when the sample approached 1,000 persons.

### Enrollment of study subjects and survey data collection

Community health workers (CHWs), most with previous experience in community-based research and familiarity with the local community, were hired for recruiting participants and survey data collection. Details of the field operations for the MAP study are described elsewhere [29]. One CHW was responsible for recruiting, obtaining informed consent, and using GPS to locate each home. Household contact forms were used to collect demographic information and respiratory health status for each member of the household. Data from these forms were entered into a database for tracking response and participation rates. Following the initial agreement to participate and the setting of appropriate times to interview, four CHWs were assigned to participating households to administer questionnaires and collect spirometry. Community Health Workers (CHWs) were trained in collection of lung function data using a portable hand-held ndd Easy on-PC Spirometry System (ndd Medical Technologies Inc. Andover, MA, USA). After the CHW determined the procedure was not contraindicated by a stroke or myocardial infarction in the past 30 days, the technique was explained and the participant seated. A minimum of three trials was attempted by the participant, with coaching by the CHW. The team pulmonologist reviewed each spirogram for reliability and repeatability using American Thoracic Society/European Respiratory Society (ATS/ERS) grading criteria. Further details regarding the procedures used in the collection of the spirometry are provided in May et al. [ 29].

CHWs used REDCap survey software on *Ipads* for all data collection. *REDCap* (Research Electronic Data Capture) is a secure, web-based software platform designed to support data capture for research studies, providing 1) an intuitive interface for validated data capture; 2) audit trails for tracking data manipulation and export procedures; 3) automated export procedures for seamless data downloads to statistical packages, and 4) procedures for data integration and interoperability with external sources [35]. The *REDCap* database, stored and backed up on servers, was exported to SAS datasets. Participants received $40 for survey completion.

The survey, which took approximately 40 min to administer, focused on established and potential risk factors for respiratory health outcomes and obtained data on current and past symptoms of respiratory health over the past 2 and 12 months before the survey. Questions were drawn primarily from established questionnaires, including the Multi-Ethic Study of Atherosclerosis (MESA) spirometry questionnaire, which provides questions similar to the National Health Interview Survey (NHIS) questions for adult asthma, and the Seattle Healthy Homes I baseline questionnaire [36–40]. Family history of respiratory disease, allergies, chronic conditions, and eczema was obtained by self-report on the questionnaire. In addition, we assessed self-reported chemical and biological triggers, focusing on environmental tobacco smoke (ETS), pesticides, VOCs, dust mites, molds, rodent and cockroach feces, and animal dander; home heating (wood, coal, gas, space heaters), home cooking (electric, wood, gas, oil), indoor smoking, pets, molds, and dampness were also assessed. Detailed information was obtained on demographic and health behavior and lifestyle factors (education, marital status, employment status, job titles, work in mining and related occupations, dietary intake, alcohol consumption, and tobacco use). At the time of the interview, pulmonary function tests were also administered to all participants who did not report a stroke or myocardial infarction in the past 30 days. At a later date, a convenience subsample of 70 participants received indoor air quality and exposure assessment to quantify fine particulates and record in-home and outdoor exposure sources. The details of pulmonary function testing and in-home air quality assessment are described in more detail elsewhere [29, 41].

### Asthma Outcomes

We examined two asthma outcomes: lifetime (“ever”) and current asthma. Lifetime asthma was coded as those responding affirmatively to the question, “Have you ever had asthma?”. Among those who reported lifetime asthma, current asthma was coded as ‘yes’ if participants reported in the past 12 months: 1) at least one asthma attack, or 2) a routine medical visit for asthma, or 3) were unable to work due to asthma, or 4) talked with a doctor or other health professional about asthma, or 5) took asthma medication, or 6) experienced any symptoms of asthma. Lifetime and current asthma variables in the analysis are based on the responses to the self-reported questions and subject to the known limitations of data without subsequent medical validation.

### Covariates

Demographic variables in the descriptive analysis included age as a three-level variable (21–34, 35–64, and 65 years and older); marital status as married/partnered or not; level of education as high school graduate (or GED) or higher; annual household income below $25,000 annually, $25,000–50,000, or greater than $50,000. Health risk behaviors examined included exposure to secondary tobacco smoke before age 16 and in adulthood; history of having smoked greater than 100 cigarettes in lifetime (never vs. ever smoker); current smoker; former smoker greater than ten years. Body mass index (BMI) was calculated as weight in pounds/height in inches^2^ multiplied by 703 and categorized as underweight < 18.5, normal 18.5–24.9, overweight 25–29.9, and obese 30 or greater. Housing type was categorized as a single-family home, mobile home, or multi-unit housing. Self-reported seasonal allergies were recorded as an indicator of atopy.

### Statistical Analysis

Frequency distributions of the demographic characteristics of the sample of respondents were calculated using Stata 12 [42]. There were relatively few missing values, so the final analysis simply omitted individuals with missing values for key variables. An exception was household income, where 24.4% of values were missing. These missing values were retained as a separate category of household income. The primary outcome variables, lifetime and current asthma were highly prevalent (> 10%) in our sample. Consequently, we used Poisson models with robust variance estimators to calculate prevalence ratios (PRs) adjusted for multiple covariates [43]. Models were adjusted for individual-level covariates (age, gender, seasonal allergies, BMI, educational attainment, type of dwelling, living with a current smoker, smoking status) that alone comprised base models, and each included a single HUC-level variable describing the intensity of a potential source of airborne particulates in the environment. Other than the base models, this resulted in ten regression analyses, one for each of the five potential sources of airborne particulates—roadways (miles per square mile), coal haul routes (miles per square mile), oil/gas wells (per square mile), abandoned mines (percent of HUC area), and active mines (percent of HUC area)—for both current and lifetime asthma. The base models were developed through consideration of significant bivariate associations observed in this study and others reported in the literature. Main effects models were fit and checked for multicollinearity and violations of other model assumptions.

## Results

### Response rates

From November 2015 to July 2017, a total of 4,291 dwellings were enumerated within 40 HUCs in the study area. From 1,459 eligible households contacted, 1,190 individual participants (82%) were recruited into the study. Of those, 218 participants did not complete the survey due to refusal, loss to follow up, or death. Of the 972 individuals recruited who completed the survey, 872 provided valid spirograms (data not presented in this paper).

### Demographic characteristics of the sample

The sample (Table 1) was primarily composed of females (59%), participants aged 35 to 64 years (61%), and those with a high school education or above (74%). Participant age ranged from 21 to 96 years with a median age of 55 years. Annual household income was reported by 76% of participants. Of these, 46% reported income less than $25,000 for the household. While most persons resided in single-family homes (65%), nearly a third lived in mobile homes, and 5% lived in multi-unit housing. Only one-fifth of our sample were employed full time, with 23% reporting being retired and 19% disabled.

**Table 1:** Characteristics study participants and distributions of cases and prevalence ratios (PR) for ever asthma and current asthma in the Mountain Air Project study (2016–2017)

	Total		Ever diagnosed w/asthma (*n* = 963)				Current asthma (*n* = 963)			
	n	%	cases	%	PR	95% CI	cases	%	PR	95% CI
All participants	972	100	218	22.6	…		157	16.3	…	
County of Residence (*n* = 972)										
Harlan	589	60.6	120	20.6	Ref	––-	81	13.9	Ref	––-
Letcher	383	39.4	98	25.6	1.24	0.98–1.57	76	19.9	1.43	1.07–1.90
Season of Interview (*n* = 972)										
Winter	192	19.8	49	25.8	1.32	0.90–1.92	35	18.4	1.66	1.00–2.74
Spring	396	40.7	92	23.6	1.20	0.86–1.69	71	18.2	1.64	1.04–2.58
Summer	195	20.1	40	20.6	1.05	0.71–1.57	30	15.5	1.39	0.83–2.34
Fall	189	19.4	37	19.6	Ref	––-	21	11.1	Ref	––-
Age group (*n* = 972)										
18–34	140	14.3	45	32.4	2.69	1.77–4.11	27	19.4	2.06	1.22–3.47
35–64	594	60.9	145	24.5	2.04	1.40–2.97	108	18.3	1.94	1.26–2.99
65 +	238	24.8	28	12.0	Ref	––-	22	9.4	Ref	––-
Gender (*n* = 972)										
Female	571	58.7	142	25.1	1.32	1.03–1.69	105	18.6	1.42	1.05–1.93
Male	401	41.3	76	19.1	Ref	––-	52	13.1	Ref	––-
Educational attainment (*n* = 971)										
< High School	254	26.1	58	23.2	1.07	0.82–1.40	48	19.2	1.33	0.96–1.83
High school or some college	599	61.6	129	21.7	Ref	––-	86	14.5	Ref	––-
College	118	12.1	30	25.4	1.17	0.83–1.65	22	18.6	1.29	0.84–1.97
Missing	1	0.1	1	100.0			1	100.0		
Annual household income (*n* = 735)										
< $25 K	451	46.4	111	24.9	1.21	0.85–1.73	82	18.4	1.22	0.79–1.88
$25-$50 K	138	14.2	32	23.5	1.15	0.74–1.78	20	14.7	0.98	0.56–1.71
$50 K +	146	15.0	30	20.5	Ref	––-	22	15.1	Ref	––-
Missing	237	24.4	45	19.1	0.93	0.62–1.41	33	14.0	0.93	0.57–1.53
Type of dwelling (*n* = 972)										
Single family house	634	65.2	134	21.3	Ref	––-	96	15.2	Ref	––-
Mobile home	288	29.6	64	22.5	1.06	0.81–1.38	43	15.1	0.99	0.71–1.38
Multi-unit housing	50	5.1	20	40.8	1.92	1.33–2.78	18	36.7	2.41	1.60–3.64
Body mass index (BMI) (kg/m^2^) (*n* = 925)										
Underweight (< 18.5)	19	2.0	5	26.3	1.35	0.61–2.96	4	21.0	1.53	0.61–3.84
Normal (18.5–24.9)	217	22.3	45	20.9	1.07	0.75–1.52	32	14.9	1.08	0.70–1.67
Overweight (25.0–29.9)	276	28.4	54	19.6	Ref	––-	38	13.8	Ref	––-
Obese (30.0 +)	413	42.5	102	25.1	1.28	0.96–1.72	75	18.4	1.34	0.93–1.92
Missing	47	4.8	12	26.1			8	17.4		
Season allergies (*n* = 969)										
Yes	546	56.2	155	28.7	1.91	1.47–2.49	113	20.9	2.00	1.44–2.76
No	423	43.5	63	15.0	Ref	––-	44	10.5	Ref	––-
Missing	3	0.3	0	0.0			0	0.0		
Smoking status (*n* = 969)										
Current	320	32.9	73	23.0	1.02	0.78–1.34	55	17.3	1.12	0.80–1.56
Former	230	23.7	49	21.9	0.98	0.72–1.32	36	15.8	1.02	0.70–1.49
Never	419	43.1	91	22.5	Ref	––-	64	15.5	Ref	––-
Missing	3	0.3	2	66.7			2	66.7		
Lives with current smoker (*n* = 972)										
Yes	626	64.4	145	23.3	1.09	0.85–1.40	106	17.1	1.14	0.84–1.56
No	346	35.6	73	21.4	Ref	––-	51	14.9	Ref	––-
Current employment (*n* = 972)										
Homemaker	194	20.0	51	26.6	2.05	1.35–3.11	39	20.3	1.91	1.18–3.08
Full-time	215	22.1	51	23.8	1.84	1.21–2.80	34	15.9	1.49	0.91–2.45
Part-time / Full-time student / Unemployed	159	16.4	33	21.0	1.62	1.02–2.57	20	12.7	1.20	0.68–2.10
Retired	219	22.5	28	13.0	Ref	––-	23	10.7	Ref	––-
Disabled	185	19.0	55	29.9	2.31	1.53–3.48	41	22.3	2.09	1.31–3.35
Ever had dusty job, including mining (*n* = 952)										
Yes	350	36.0	64	18.4	Ref	––-	46	13.3	Ref	––-
No	622	64.0	154	25.0	1.36	1.04–1.76	111	18.0	1.36	0.99–1.87

*PR* prevalence ratio

Cigarette smoking remains highly prevalent in this region, with 33% of the sample reporting current smoking while 24% were classified as former smokers; 65% of the sample reported living in the home of a current smoker as an adult. Reflecting regional health characteristics, 29% of the sample were overweight, and 42% were obese. More than one-third had been employed in a dusty job, including mining, during their working career. Among men, however, 80% had been employed in mining, compared to only 5% among women.

The overall rate of persons reporting they had ever been diagnosed with asthma was 22.8%, and the prevalence of current asthma was 16.3%. Lifetime and current asthma were most prevalent in women and among those in the youngest age group (21–34 years).

Both lifetime asthma (PR = 1.92; 95% CI: 1.33–2.78) and current asthma (PR = 2.41; 95% CI: 1.60–3.64) were roughly two-fold higher in residents of multi-unit housing compared to those in single-family homes. Those reporting seasonal allergies also had a higher prevalence of both lifetime (PR = 1.91) and current asthma (PR = 2.00) in comparison to those not reporting allergies. Homemakers, as well as those who were disabled, had a higher prevalence of lifetime asthma and current asthma compared to the retired, who had the lowest prevalence. Those who had ever worked in dusty occupations reported lower rates of lifetime and current asthma.

An unadjusted analysis of the associations between our HUC-based GIS metrics and lifetime and current asthma is displayed in Table 2 for the 963 participants with complete data. There was a significant positive dose–response relationship between roadway density (road miles/square mile) and lifetime and current asthma prevalence. For current asthma, the prevalence ratio is more than two-fold higher (PR = 2.14; 95% CI: 1.43–3.22) at the highest tertile of roadway density. The rates of both lifetime and current asthma appeared to increase with greater density of oil and gas wells in the hollow, but the associations were not significant. The prevalence rates of lifetime and current asthma were lowest at the highest tertiles of the intensity of active mining (measured as a percent of the hollow area) in this unadjusted analysis.

**Table 2:** Prevalence of ever asthma and current asthma by geographic exposure metrics among study participants in the Mountain Air Project study (2016–2017)

	Ever diagnosed w/asthma (*n* = 963)				Current asthma (*n* = 963)			
	n	%	PR	95% CI	n	%	PR	95% CI
Road miles/sq. mi								
Tertile 1 (0.19–1.89)	49	16.2	1.0	–-	29	9.6	1.0	–-
Tertile 2 (2.18–2.76)	75	23.0	1.42	1.03–1.96	59	18.1	1.88	1.24–2.86
Tertile 3 (2.96–6.00)	94	28.1	1.73	1.27–2.35	69	20.6	2.14	1.43–3.22
Coal haul miles/sq. mi								
Zero	66	21.9	1.0	–-	50	16.6	1.0	–-
Below median (0.00–0.62)	108	22.4	1.02	0.78–1.34	81	16.8	1.01	0.73–1.40
Above median (0.72–1.27)	44	24.4	1.11	0.80–1.56	26	14.4	0.87	0.56–1.35
Oil/gas wells/sq. mi								
Zero	50	20.8	1.0	–-	32	13.3	1.0	–-
Below median (0.34–3.51)	132	22.5	1.09	0.81–1.45	96	16.7	1.26	0.87–1.82
Above median (4.38–8.44)	36	26.5	1.28	0.88–1.85	27	19.9	1.50	0.94–2.39
Abandoned mining, %HUC area								
Tertile 1 (0.00–8.04)	65	21.2	1.0	–-	45	14.7	1.0	–-
Tertile 2 (9.72–44.84)	77	22.1	1.04	0.78–1.40	53	15.2	1.04	0.72–1.49
Tertile 3 (46.40–196.45)	76	24.6	1.16	0.87–1.55	59	19.1	1.30	0.91–1.85
Active mining, %HUC area								
Zero	113	25.3	1.0	–-	84	18.8	1.0	–-
Below median (0.00–8.02)	70	23.0	0.91	0.70–1.18	50	16.4	0.87	0.63–1.20
Above median (9.68–48.31)	35	16.5	0.65	0.46–0.92	23	10.9	0.58	0.37–0.89

*PR* prevalence ratio

Adjusted models—the base models—using the robust Poisson regressions are shown in Table 3. The adjusted prevalence ratios (aPR) indicate significantly higher lifetime and current asthma prevalence in the youngest age group (18–34 years) compared to those over age 65 and significantly higher prevalence of current asthma in women (aPR = 1.40, p = 0.04). Seasonal allergies and residing in a multi-unit housing complex (in comparison to single-family housing) remained significant risk factors for both lifetime and current asthma in the base models. Current asthma was 50% (aPR = 1.50; 95% CI: 1.09–2.07) more prevalent among those with less than a high school education in the model.

**Table 3:** Base model for lifetime and current asthma

	Lifetime asthma (*n* = 910)			Current asthma (*n* = 910)		
	aPR	*P*-value	95% CI	aPR	*P*-value	95% CI
Age group						
18–34	2.83	< 0.001	1.77–4.51	2.04	0.01	1.15–3.62
35–64	2.10	< 0.001	1.42–3.12	1.90	0.01	1.21–2.97
65 +	Ref		––-	Ref		––-
Gender						
Female	1.21	0.14	0.94–1.56	1.40	0.04	1.02–1.93
Male	Ref		––-	Ref		––-
Seasonal allergies						
Yes	1.86	< 0.001	1.42–2.43	1.91	< 0.001	1.37–2.67
No	Ref		––-	Ref		––-
Body mass index (BMI) (kg/m^2^)						
Underweight (< 18.5)	1.25	0.55	0.60–2.57	1.37	0.47	0.59–3.22
Normal (18.5–24.9)	1.04	0.83	0.73–1.48	1.00	0.99	0.64–1.56
Overweight (25.0–29.9)	Ref		––-	Ref		––-
Obese (30.0 +)	1.17	0.30	0.87–1.57	1.22	0.27	0.85–1.76
Educational attainment						
< High School	1.19	0.21	0.90–1.58	1.50	0.01	1.09–2.07
High school or some college	Ref		––-	Ref		––-
College	1.34	0.11	0.94–1.93	1.47	0.10	0.93–2.34
Type of dwelling						
Single family house	Ref		––-	Ref		––-
Mobile home	1.02	0.87	0.77–1.36	0.98	0.89	0.69–1.38
Multi-unit housing	1.71	0.01	1.13–2.57	2.01	0.003	1.27–3.18
Lives with current smoker						
Yes	1.23	0.22	0.89–1.70	1.20	0.39	0.79–1.83
No	Ref		––-	Ref		––-
Smoking status						
Current	Ref		––-	Ref		––-
Former	1.26	0.18	0.90–1.77	1.16	0.46	0.78–1.73
Never	1.28	0.16	0.91–1.81	1.12	0.60	0.73–1.72

*PR* prevalence ratio

Table 4 provides prevalence ratio estimates for each of the environmental exposure metrics and lifetime and current asthma adjusted for all variables in the base models (i.e., Table 3). Roadway density (road miles /square mile) was positively associated with current asthma when comparing the second tertile (aPR = 1.61; 95% CI:1.04–2.48) and third tertile (aPR = 2.00; 95% CI:1.32–3.03) to the lowest first tertile of road miles/square mile. For lifetime asthma, roadway density was positively associated with asthma prevalence, with a significant 56% increased prevalence at the third tertile. For current asthma, there was some elevation in prevalence (aPR = 1.70) among those living in areas with oil and gas well density above the median. Adjusted prevalence ratios for lifetime and current asthma were lowest at the above-median levels of active mining density within the HUC, with statistically significant protective ratios for lifetime (aPR = 0.64) and current (aPR = 0.54) asthma.

**Table 4:** Adjusted prevalence ratios for lifetime and current asthma by environmental exposure

	Lifetime asthma (*n* = 910)			Current asthma (*n* = 910)		
	aPR	*P*-value	95% CI	aPR	*P*-value	95% CI
Road miles/sq. mi						
Tertile 1 (0.19–1.89)	Ref		––-	Ref		––-
Tertile 2 (2.18–2.76)	1.22	0.24	0.88–1.71	1.61	0.03	1.04–2.48
Tertile 3 (2.96–6.00)	1.56	0.01	1.14–2.14	2.00	0.001	1.32–3.03
Coal haul miles/sq. mi						
Zero	Ref		––-	Ref		––-
Below median (0.00–0.62)	0.87	0.34	0.66–1.15	0.84	0.30	0.60–1.17
Above median (0.72–1.27)	1.03	0.88	0.73–1.44	0.79	0.30	0.50–1.24
Oil/gas wells/sq. mi						
Zero	Ref		––-	Ref		––-
Below median (0.34–3.51)	1.04	0.81	0.76–1.43	1.26	0.28	0.83–1.91
Above median (4.38–8.44)	1.30	0.19	0.88–1.92	1.70	0.04	1.03–2.80
Abandoned mining, %HUC area						
Tertile 1 (0.00–8.04)	Ref		––-	Ref		––-
Tertile 2 (9.72–44.84)	1.01	0.96	0.74–1.37	0.94	0.76	0.64–1.38
Tertile 3 (46.40–196.45)	1.18	0.28	0.88–1.58	1.31	0.14	0.91–1.89
Active mining, %HUC area						
Zero	Ref		––-	Ref		––-
Below median (0.00–8.02)	0.87	0.33	0.66–1.15	0.78	0.16	0.56–1.10
Above median (9.68–48.31)	0.64	0.02	0.45–0.92	0.54	0.01	0.35–0.85

*aPR*adjusted prevalence ratio

Prevalence ratios adjusted for variables in base model, but not other environmental exposures

## Discussion

The prevalence estimates of lifetime (22.8%) and current asthma (16.3%) in the MAP sample affirm the high prevalence documented in previous work among rural Appalachian populations [15]. Our prevalence estimates are subject to a small upward bias for asthma (see below) since our methods of enrolling persons intentionally favored the selection of persons with any respiratory health outcome (COPD, asthma, other lung disease) into the study. While this approach increased the likelihood of recruiting those with respiratory health conditions beyond a random sample of eligible adults in the household, the approach provided a more robust analysis of the associations of the environmental exposures with asthma.

Current smoking, exposure to environmental tobacco smoke, and obesity rates are significantly higher in this sample than in the US population and may account, in part, for high asthma prevalence in this geographic area. The paper by Mabila et al. with the NHI survey affirms the strong association of respiratory disease, including asthma, in men working in the dusty trades industries [44]. In the MAP cohort, 69% of men had worked in coal mining occupations, including above and underground, as well as MTR. Consequently, the ability to separate the occupational component from environmental exposure is challenging, especially in men. Only 15 women in our study reported working in mining or other dusty trades. The asthma prevalence in this study is higher in the younger age and in the lower exposure groups among men in the mining industry. Another bias not often mentioned is the healthy smoker effect, whereby those with better lung function may be more likely to be smokers than persons initially with asthma [45]. The longitudinal data examining the association between smoking and asthma are definitive; associations between smoking and asthma are less strong in cross-sectional data.

### Roadway density

The positive associations of lifetime and current asthma with increasing roadway density, measured at the level of the HUC in the adjusted analysis, is consistent with other literature that examined asthma in relation to traffic density, particularly in urban areas [3, 4]. In our analyses, the association with roadway density was the strongest and most consistent finding, following adjustment for other known risk factors. Our finding of a strong association between roadway density and risk for lifetime and current asthma in a rural mining community is notable and requires further confirmation.

The majority of studies of traffic-related air pollutants (TRAP) and asthma have been undertaken in urban environments and focused primarily on childhood asthma. Porebski et al. (2014) has examined the relationship between current asthma symptoms in children in Poland and distance to major roadways, with symptom prevalence being greatest for those living less than 200 m from the roadway [46]. A study among 6,399 adults from the Framingham cohort found that living close to a major roadway (less than 400 m) was associated with an increased prevalence of adult asthma with an adjusted odds ratio of 1.35 (95% CI: 1.06–1.72) for those living 200-less than 400 m away in comparison to those living greater than 400 m from a roadway. A study by Rice in among participants of the Framingham Offspring and Third Generation studies showed the association between traffic-related air pollution and changes in lung function decline over relatively short distances and with relatively low levels of air pollutants [47]. Lindgren et al. reported significant associations between asthma exacerbations and residential proximity to traffic in a cohort of adults and children in Minnesota [48]. Generally, the higher levels of TRAP, which are associated with roadway proximity or density, reflect exposure to particulates, nitrogen oxide, diesel, carbon monoxide, sulfur oxide, and other volatile organic hydrocarbons [49].

There are multiple components of traffic-related emissions that are likely involved in influencing asthma, which is itself a heterogeneous condition. In addition to nitrogen oxides, sulfur oxides, and other volatile organic hydrocarbons, traffic emissions are composed of fine, ultrafine, and nanoparticles which have recently been shown to be among the most hazardous particles [48]. Traffic and proximity to roadways may be associated with other exposures, including noise. Metrics such as traffic density and distance to roadways may better measure the cumulative effects and various components of TRAP exposures rather than considering any one component individually. Gowers et al. provide a review of potential mechanisms including oxidative stress, airway remodeling, and inflammation and sensitization by which TRAP may induce new cases of asthma as well as exacerbate the symptoms of asthma [50]

Our study is unique in highlighting the effect of roadway density in a rural area in eastern Kentucky on adult asthma. There are few EPA air pollution monitoring stations in these areas, and consequently, there are limited data for PM levels in these rural areas compared to more intensively monitored urban areas. Geographic characteristics of these rural areas may trap pollutants, perhaps during cold inversions events, and enhance PM levels [41]. The type of housing (see below) may also be a factor influencing the levels of indoor pollutants, in addition to lifestyle factors such as the amount of time rural residents spend at home and spend in outdoor locations, such as porches, driveways, and yards.

### Public housing

In multivariable modeling, residence in multi-unit housing was a significant predictor of both lifetime asthma and current asthma. Apartments included in the sample were almost exclusively public or subsidized housing. This finding is consistent with previous studies focused on children living in large public housing facilities in urban areas [36, 38, 51]. Our findings suggest that residence in public housing may also be an independent contributor to differences in asthma prevalence in rural adults. In an extensive review of the literature, Mendell et al. found sufficient evidence for an association between dampness and mold and asthma development, current asthma, and ever asthma [52]. Walkthrough data from the homes and anecdotal observation from our study indicated that dampness was prevalent in many of the homes [41]. Their suggestion was that indoor dampness and mold prevention are likely to reduce the risk of asthma even without consideration of the specific microbiologic agents involved.

### Mining and other GIS metrics

While we found a high prevalence of current asthma (16.3%) among adults in the MAP study, consistent with a previous cross-sectional study by Hendryx et al. (2013), no significant positive associations between current or lifetime asthma prevalence and increasing levels of active coal mining within the HUCs were found in multivariable analysis. Rather, we observed a significant protective effect associated with the highest tertile of active mining. Roadway density—a metric more closely associated with current and lifetime asthma in adjusted analyses—was significantly negatively correlated with active mining, however, which suggests an explanation for this counterintuitive finding. Furthermore, during the period of data collection, few active surface mines remained in the study area, yielding only 35 participants with asthma who resided within above-median active mining surface areas.

It is important to consider the average duration of residence in the interpretation of the results. Long-time residents may have had asthma due to dusty conditions in the past. The average duration of residence for persons in the entire study was 17.2 years. If mining-related dusts are one potential causal factor related to asthma incidence, or if they trigger asthma episodes, then one may have expected higher prevalence ratios with closer proximity to these mining sites. Further, we do not have data on particulate levels in the past, which may have been much higher, from the local coal mining operations [41]. Finally, the mining operations in the area, especially the surface and mountaintop removal mines, may have only been operational for a limited time in the past, and dust-related exposures due to blasting, coal transport, and active mining operations may have been of relatively limited duration.

While our small area metrics of potential exposures at the level of the HUC to mining dust or other mining-related contaminants are limited, they likely represent an improvement to earlier studies that simply characterized whole counties by a mining status classification [18, 19, 21]. Our HUC-based metrics characterize potential exposures at a neighborhood level with a median HUC size of less than 1.8 square miles. There is the potential for exposure misclassification with our strategy, which assigned exposure values at the level of the HUC for roadway density and coal haul routes (road miles/square mile), active and abandoned mining sites (percent of HUC total surface area), and oil and gas wells (wells/square mile). Unknown variables such as the location of dust-producing activities within the permitted mine area, the operational status of facilities, wind directions, length of time in operation (open and closing dates), size of the mining facility, type of mining, and adherence to regulations are all factors which impact PM levels within small areas. However, recent work by our group does indicate a strong concordance between the levels of PM2.5 measured at residences and the HUC estimates of the tertiles of roadway density. In the case of roadway density, our study created a substantial range in exposure variability, which may have allowed for the detection of an effect, whereas our data on mining activity was more limited, with a smaller proportion of HUCs having "high mining exposure." It would be a reasonable expectation that our exposure classification using HUCs was nondifferential by adult self-reported asthma status.

### Strengths and limitations

This community-based study was undertaken in two counties in Kentucky which provided geographic diversity and exposure to both active and historic coal mining operations and documented a high prevalence of respiratory health outcomes. The enumeration and recruitment occurred through direct door-to-door contact. In rural community studies of adults, using community-based interviewers is essential to securing participation and gaining a high level of completeness for the questionnaires. Community members were engaged and supportive of a study to address these issues. The overall response rate for eligible households in this study was 82%, and we obtained relatively complete data collection from the face-to-face surveys due to well-trained community-based interviewers and diligent effort [29]. We further note that 28 of 40 HUCs enumerated had recruitment rates of 80% or greater and five HUCs had recruitment rates exceeding 90% which reduced issues with potential selection bias across the small geographic areas (HUCs) [29].

Our sampling approach prioritized enrolling those who reported respiratory illness. Such an approach may have introduced a selection bias, potentially inflating the prevalence estimates above what may have occurred in a random sample. This would occur in the situation where there were two or more adults in the home, and at least one adult did not have a respiratory health outcome and the other did and agreed to participate. This would lead to a small increase in the prevalence of respiratory disease in the sample relative to the eligible population. For current asthma, we estimate a 3% increase in asthma prevalence in our enrolled sample in comparison to the fully enumerated sample (all household members) in the study. Although the prevalence of the outcome may be overestimated, the magnitude of the effect estimates for the predictors of asthma outcomes in the Poisson models, where the effect estimates are adjusted prevalence ratios, should not be affected by the sampling approach that we used. While our sample provided good representation across education and income levels which are comparable to Census estimates, we do note that we had greater participation from women compared to men and those persons who were more likely to be home at the time of contact with our enumerators when comparing the enrolled sample to the fully enumerated sample frame in the study [29].

This cross-sectional study has the standard limitations of being conducted during a single time period and obtaining most of the health outcome and covariate data by self-report on the interviewer-administered questionnaire. Computer-assisted data entry using iPads limited the amount of missing data from the questionnaire. The enrollment of adults from eligible households has the potential for selection bias in that recruitment required a person was at the household during one of the multiple attempts to make contact. For example, persons who may have worked in the evenings or weekends or spent little time at home may not have been available for inclusion. There was no sampling frame for all occupied households or a phone listing in these communities, such as a 911 emergency listing for use in the study. Details of the enumeration and survey approach are provided elsewhere [29].

The population of adults residing in these two counties has been relatively stable; we report a duration of residence of 17 years among study participants in their current homes. However, since 2010 there has been substantial outmigration of segments of the population, with an estimated 9% abandoned dwellings estimated by our field staff during the enumeration survey. This result has been supported by a recent Census projection of about a 10% loss to the population in this area. Those who stay and who participated in the survey have been there for a relatively long time, but a segment of the younger population no longer resides in these communities, and they are communities with an older structure to their age pyramids.

A series of reports by Hendryx et al. and others have attracted substantial attention for documenting associations, generally at the county level in ecologic analysis, between living in areas with active surface or mountaintop removal mining and a range of health outcomes, including mortality and morbidity from respiratory diseases such as asthma and COPD [18–21, 23–25]. There has been an ongoing debate regarding whether the associations between mining related exposures, typically residence within a county with high coal production or residence near mining locations, and respiratory disease outcomes are likely causal or due to confounding or other biases. There are biologically plausible reasons to infer that exposures from mining including mineral dusts, metals, diesel, air pollutants (NO, SO_2_, CO) may be biologically related to respiratory impairments. In addition, the two counties in our study are located within the Central Appalachian region – well known as some of the highest areas of unemployment, poverty, lower educational attainment, and poor health status.

The limitations of ecologic studies for individual level inferences are well known and, in particular, the limitations of the series of studies, primarily among Appalachian mining communities in West Virginia and Kentucky, have been discussed in detail by Borak et al. [26]. In general, the inferences made at the ecologic level are not comparable to those made at the individual level. Among the several critiques directed at the previous studies, the limitations with the exposure assessment, typically county level proxies of mining activity, the lack of individual level and complete covariate adjustment, concerns regarding missing variables, and selection biases are the most concerning. For most of these economically distressed counties in Appalachia, there is a substantial overlay of lifestyle risk factors (poverty, unemployment, lower educational levels, poor health status, smoking, and obesity) and potential exposures from coal mining, proximity to roadways, and other sources of contaminants that may impact respiratory function. For successful statistical control of these variables, it is required that there is a differential distribution of each across the units of comparison, whether counties or individuals, to detect effects. The ecologic study design is insufficient to address the question of the individual level health outcomes from a complex mixture or exposure and lifestyle variables.

The MAP study provides small area estimates of exposures, at the level of the hydrologic unit classification, to surface and underground mining, traffic density, coal haul routes, and oil and gas operations. This allowed for a range of these exposures within the county and assigned at the individual level, along with individual level covariates for current smoking, environmental tobacco smoke, occupational exposures, and other lifestyle risk factors. The variation of these exposures within participants in our study contrasts with the assignment of only a county-level exposure variable in most previous research. Our efforts to enhance the sampling of persons throughout the two counties by different geographic regions and in relation to their proximity to diverse sources of exposure improves on studies which have employed convenience samples.

## Conclusion

Our data suggest that proximity to roadways and conditions of public housing, in particular, increase the asthma risk among adults. Current exposures to dust from mining operations may have influenced respiratory outcomes in the past, but we found no evidence of an association with the small area metrics that we used for the environmental exposures related to mining and oil and gas operations. From the public health perspective, a focus on continuing efforts to reduce exposure to ETS in homes may have the largest impact on the reduction of adult asthma. Well-established risk factors for asthma incidence and episodes, such as ETS, current smoking, and dampness, should be the focus of intervention efforts in these communities. Future research with the MAP data will more closely examine asthma episodes, the heterogeneity of the disease, use of medication, and exposure to PM 2.5.

## Data Availability

The datasets used and/or analyzed during the current study are available from the corresponding author on reasonable request.
